# Necessity of Routinely Testing the Proximal and Distal Ends of Exposed Recurrent Laryngeal Nerve During Monitored Thyroidectomy

**DOI:** 10.3389/fendo.2022.923804

**Published:** 2022-06-30

**Authors:** Hsiao-Yu Huang, Ching-Feng Lien, Chih-Chun Wang, Chien-Chung Wang, Tzer-Zen Hwang, Yu-Chen Shih, Che-Wei Wu, Gianlorenzo Dionigi, Tzu-Yen Huang, Feng-Yu Chiang

**Affiliations:** ^1^ Department of Otolaryngology-Head and Neck Surgery, E-Da Hospital, Kaohsiung, Taiwan; ^2^ School of Medicine, College of Medicine, I-Shou University, Kaohsiung, Taiwan; ^3^ Department of Otolaryngology, E-Da Cancer Hospital, Kaohsiung, Taiwan; ^4^ Department of Otorhinolaryngology-Head and Neck Surgery, International Thyroid Surgery Center, Kaohsiung Medical University Hospital, Faculty of Medicine, College of Medicine, Kaohsiung Medical University, Kaohsiung, Taiwan; ^5^ Division of General Surgery, Endocrine Surgery Section, Istituto Auxologico Italiano Istituto di Ricovero e Cura a Carattere Scientifico (IRCSS), Milan, Italy; ^6^ Department of Pathophysiology and Transplantation, University of Milan, Milan, Italy

**Keywords:** recurrent laryngeal nerve (RLN), intraoperative neuromonitoring (IONM), thyroid surgery, electromyography (EMG), vocal cord (VC) mobility

## Abstract

**Objectives:**

Intraoperative neuromonitoring (IONM) is a useful tool to evaluate the function of recurrent laryngeal nerve (RLN) in thyroid surgery. This study aimed to determine the necessity and value of routinely testing the proximal and distal ends of RLN.

**Methods:**

In total, 796 patients undergoing monitored thyroidectomies with standardized procedures were enrolled. All 1346 RLNs with visual integrity of anatomical continuity were routinely stimulated at the most proximal (R2p signal) and distal (R2d signal) ends after complete RLN dissection. The EMG amplitudes between R2p and R2d signals were compared. If the amplitude of R2p/R2d ratio reduction (RPDR) was over 10% or loss of signal (LOS) occurred, the exposed RLN was mapped to identify the injured point. Pre- and post-operative vocal cord (VC) mobility was routinely examined with video-laryngofiberscope.

**Results:**

Nerve injuries were detected in 108 (8%) RLNs, including 94 nerves with incomplete LOS (RPDR between 13%-93%) and 14 nerves with complete LOS. The nerve injuries were caused by traction in 80 nerves, dissecting trauma in 23 nerves and lateral heat spread of energy-based devices in 5 nerves. Symmetric VC mobility was found in 72 nerves with RPDR ≤50%. The occurrence of abnormal VC mobility (weak or fixed) was 14%, 67%, 100%, and 100% among the different RPDR stratifications of 51%-60%, 61%-70%, 71%-80%, and 81-93%, respectively. Of the 14 nerves with complete LOS, all showed fixed VC mobility. Permanent VC palsy occurred in 2 nerves with thermal injury.

**Conclusion:**

Routinely testing the proximal and distal ends of exposed RLN helps detect unrecognized partial nerve injury, elucidate the injury mechanism and determine injury severity. The procedure provides accurate information for evaluating RLN function after nerve dissection and should be included in the standard IONM procedure.

## Introduction

Intraoperative neuromonitoring (IONM) has been commonly applied in thyroid surgery to facilitate the identification of recurrent laryngeal nerve (RLN), evaluate nerve function after dissection, detect nerve injury and elucidate its mechanism, predict the outcome of vocal cord (VC) function and make the decision to perform staged thyroidectomy (1–6).

From the literature review, the era in the absence of standard IONM procedures revealed a high negative predictive value of 92%-100%, but a low and highly variable positive predictive value, ranging from 10% to 90% (7–13). The results suggested that patients with positive EMG signals after thyroid resection generally do not have VC palsy. Conversely, patients with loss of signal (LOS) have unpredictable VC functional outcomes, leading to unnecessary staged operation.

In recent years, through following the standard 4-step IONM procedures (V_1_-R_1_-R_2_-V_2_) and troubleshooting algorithms, the positive predictive value has greatly improved. When LOS occurs after RLN dissection, it always indicates nerve injury and the development of postoperative VC palsy (5, 6, 14–16). However, a positive EMG signal at the proximal end of RLN does not guarantee normal nerve function and normal postoperative VC function. In the absence of complete LOS, a partial nerve injury could be unrecognized and an unexpected VC palsy may occur (17, 18). In this study, we hypothesize that routinely testing the proximal and distal ends of exposed RLN may help detect unrecognized nerve injury, elucidating the injury mechanism, and determining injury severity.

## Materials and Methods

### Patients

From January 2015 to October 2019, 796 patients (152 men and 644 women; ages ranging from 12 to 81 years; mean age, 50.4 years) underwent operations for various thyroid diseases by the same surgeon (F.-Y. C) were collected. There were 232 thyroid lobectomies and 564 total thyroidectomies (467 benign and 329 malignant thyroid diseases). Fourteen nerves were excluded from this study due to preoperative cord palsy (10 nerves) and intentional sacrifice due to cancer encasement (4 nerves). Thus, 1346 nerves at risk were enrolled in this study. The study was approved by the Institutional Review Board (IRB) of Kaohsiung Medical University Hospital, Taiwan: KMUHIRB-E(II)-20200348.

### IONM Setup and Procedures

The standard procedures for equipment setup and anesthesia were performed by the IONM team of Kaohsiung Medical University Hospital. All patients were intubated with a regular oral endotracheal tube. After dissection of the thyroid pyramidal lobe, two paired subdermal needle electrodes (length 12.0 mm, Medtronic Xomed, Jacksonville, FL, USA) were obliquely inserted into the sub-perichondrium of the lateral thyroid cartilage on each side for EMG signal recording (19, 20).

During the operation, standardized IONM procedures were strictly followed. The VN (without dissecting the carotid sheath to expose the VN) was stimulated with a 5-10 mA stimulus current before and after RLN dissection, and V_1_ and V_2_ signals were obtained. The RLN was stimulated with 3-5 mA at the first identification, and the R_1_ signal was obtained. After complete RLN dissection, the exposed RLN was stimulated at the most proximal end, and distal end near the laryngeal entry point, and R_2p_ and R_2d_ signals were obtained, respectively. The largest amplitudes of these five EMG signals (V_1_-R_1_-R_2p_-R_2d_-V_2_) were registered in all cases. The amplitudes of the R_2p_ and R_2d_ signals were compared. If the amplitude of R_2p_/R_2d_ ratio reduction (RPDR) was over 10% after repeated testing or LOS occurred, the whole exposed RLN was mapped to identify the weak or disrupted point of nerve conduction with 1 mA. The standardized IONM techniques in this study started with a preoperative examination of VC function (L_1_), and an intraoperative 5-step procedure (V_1_-R_1_-R_2p_-R_2d_-V_2_), and ended with a postoperative examination of VC function (L_2_) for all patients (**Table 1**).

**Table 1:** Standardized IONM procedures.

Procedures
**L_1_** Pre-operative examination of VC function with laryngofiberscope
**V_1_** Vagus nerve stimulation with 5-10 mA before thyroid dissection
**R_1_** RLN stimulation with 3-5 mA at first RLN identification
(R_2p_)* RLN stimulation with 3-5 mA at the most proximal end after RLN dissection
(R_2d_)* RLN stimulation with 3-5 mA at the distal end near the laryngeal entry point
**V_2_** Vagus nerve stimulation with 5-10 mA after resection of thyroid lobe
**L_2_** Post-operative examination of VC function with laryngofiberscope

*RLN mapping with 1 mA if the amplitude reduction of R_2p_/R_2d_ signals over 10% or LOS

IONM, intraoperative neuromonitoring; RLN, recurrent laryngeal nerve; VC, vocal cord.

For the severity of nerve injury, complete LOS was defined as an absence of EMG signals (R_2p_ amplitude less than 100 µV) after nerve stimulation. Incomplete LOS was defined as when the R_2p_ amplitude was detectable (more than 100 µV), the RDPR was more than 10% and less than 100%. When nerve injury occurred, the mechanism of nerve injury (traction injury, dissecting trauma and thermal injury) and injury site (upper third, middle third and lower third portion of the exposed RLN) were registered. For the type of nerve injury, type 1 LOS was defined as a detectable injury site on the exposed RLN, and type 2 LOS was defined as no detectable injury site on the whole exposed RLN. For VC mobility, normal (symmetric) VC function was correlated with symmetric VC mobility during phonation, weak VC function was correlated with weak VC mobility (asymmetric and sluggish VC movement at the injury side), and VC palsy was correlated with fixed VC mobility. When VC palsy persisted for more than 6 months, it was regarded as permanent palsy.

The percentage and mean value were calculated using Microsoft Excel 2016 (Microsoft Corp., Redmond, WA, USA).

## Results

Among 1346 RLNs with anatomical integrity in continuity after nerve dissection, nerve injury was detected in 108 (8%) nerves, including 94 nerves with incomplete LOS (RPDR ranging from 13% to 93%) and 14 nerves with complete LOS.

Of the 94 RLNs with incomplete LOS, the nerve injury site was detectable in all nerves. Seventy-three nerve injuries were caused by traction injury, 19 nerve injuries were caused by dissecting trauma, and 2 nerve injuries were caused by thermal injury. For injured nerves with RPDR ≤50%, the 72 corresponding patients had postoperative symmetric VC mobility; for injured nerves with RPDR between 51%-60%, weak VC mobility was found in 1 (14%) of 7 corresponding patients; for injured nerves with RPDR between 61%-70%, abnormal (2 weak and 2 fixed) VC mobility was found in 4 (67%) of 6 corresponding patients; for injured nerves with RPDR between 71%-80%, abnormal (1 weak and 2 fixed) VC mobility was found in 3 (100%) of 3 corresponding patients; for injured nerves with RPDR between 81%-93%, abnormal VC mobility was found in 6 (100%) of 6 corresponding patients, and all patients had fixed VC mobility (**Tables 2**, **3**).

**Table 2:** The mechanism and severity of RLN injury and the outcome of VC mobility in 108 RLNs detected with injury.

Severity of nerve injury	Case number	Mechanism of nerve injury and injury site			VC mobility		
		Traction injury (U/M/L)	Dissecting trauma (U/M/L)	Thermal injury (U/M/L)	Sym	Weak	Fixed
Incomplete LOS (RPDR >10%, <100%)	94	73(73/0/0)	19(2/6/11)	2(0/1/1)	80	4	10
11%-20%	35	32	3	0	35	0	0
21%-30%	16	12	3	1	16	0	0
31%-40%	10	6	4	0	10	0	0
41%-50%	11	7	4	0	11	0	0
51%-60%	7	7	0	0	6	1	0
61%-70%	6	5	1	0	2	2	2
71%-80%	3	1	2	0	0	1	2
81%-90%	3	1	1	1	0	0	3
91%-93%	3	2	1	0	0	0	3
Complete LOS	14	7(4/0/0)^+^	4(1/2/1)	3(3/0/0)	0	0	14*
Total	108	80	23	5	80	4	24

^+^type 1 complete LOS in 4 nerves with detectable injured point, type 2 complete LOS in 3 nerves without detectable injured point.

*temporary palsy in 12 nerves, permanent palsy in 2 nerve with thermal injury.

U/M/L, upper/middle/lower portion of the exposed RLN.

RLN, recurrent laryngeal nerve; VC, vocal cord; RPDR, R_2p_/R_2d_ ratio reduction; LOS, loss of signal; sym, symmetric.

**Table 3:** Intraoperative EMG signals and mechanism of nerve injury in the RLNs without complete LOS (detectable injury site and R_2p_/R_2d_ reduction >50%).

No.	V_1_ (µV)	R_1_ (µV)	R_2p_ (µV)	R_2d_ (µV)	V_2_ (µV)	R_2p_/R_2d_ reduction	Injury site	Injury mechanism	VC mobility
1	949	1017	708	1431	655	51%	U(BL)	traction	sym
2	2051	2215	1188	2491	1037	52%	U(BL)	traction	sym
3	3598	3752	1337	2806	1068	52%	U(BL)	traction	sym
4	2728	2899	1393	3110	979	55%	U(BL)	traction	sym
5	1261	1358	512	1214	358	58%	U(BL)	traction	weak
6	1570	2237	989	2441	606	59%	U(BL)	traction	sym
7	1957	2354	954	2325	801	59%	U(BL)	traction	sym
8	2235	3695	1480	3855	1072	62%	U(BL)	traction	weak
9	1153	1278	364	1480	244	66%	U(BL)	traction	sym
10	921	843	396	1230	272	68%	U(BL)	traction	fixed^t^
11	752	780	229	724	167	68%	L	trauma	fixed^t^
12	2707	4570	1647	5254	903	69%	U(BL)	traction	sym
13	1175	1363	688	2264	462	70%	U(BL)	traction	weak
14	1728	3494	880	3422	543	74%	U(BL)	traction	weak
15	1889	2206	509	2144	577	76%	L	trauma	weak
16	1287	1658	346	1712	247	80%	M	trauma	fixed^t^
17	1152	1289	145	755	138	81%	U(BL)	traction	fixed^t^
18	1564	1735	351	1837	287	81%	L	thermal	fixed^t^
19	1308	1521	188	1810	122	90%	L	trauma	fixed^t^
20	2896	3015	250	3109	235	92%	U(BL)	traction	fixed^t^
21	2779	3410	323	3822	190	92%	U(BL)	traction	fixed^t^
22	2935	3773	232	3351	217	93%	U(BL)	trauma	fixed^t^

EMG, electromyography; RLN, recurrent laryngeal nerve; LOS, complete loss of signal; VC, vocal cord; U(BL), upper portion of the exposed RLN (at the region of Berry’s ligament); M, middle portion of the exposed RLN; L, lower portion of the exposed RLN; sym, symmetric; fixed ^t^, temporary palsy.

Of the 14 RLNs with complete LOS, the nerve injury site was detectable on the exposed RLN in 11 nerves (type 1 LOS) and was not detectable on the exposed RLN in 3 nerves (type 2 LOS). Seven nerve injuries were caused by traction injury, 4 nerve injuries were caused by dissecting trauma, and 3 nerve injuries were caused by thermal injury. All 14 cases showed fixed VC mobility postoperatively. Temporary VC palsy occurred in 12 nerves, and permanent VC palsy occurred in 2 nerves with thermal injury (**Tables 2**, **4**).

**Table 4:** Intraoperative EMG signals and mechanism of nerve injury in the RLNs with complete LOS.

No.	V_1_ (µV)	R_1_ (µV)	R_2p_ (µV)	R_2d_ (µV)	V_2_ (µV)	R_2p_/R_2d_ reduction	Injury site	Injurymechanism	VC mobility
1	2862	2993	LOS	3279	LOS	=100%	U(BL)	traction	fixed^t^
2	1391	1581	LOS	1242	LOS	=100%	U(BL)	traction	fixed^t^
3	2430	2882	LOS	2976	LOS	=100%	U(BL)	traction	fixed^t^
4	3616	4600	LOS	5019	LOS	=100%	U(BL)	traction	fixed^t^
5	1716	2585	LOS	LOS	LOS	=0%	nil	traction	fixed^t^
6	2145	2521	LOS	LOS	LOS	=0%	nil	traction	fixed^t^
7	1053	1384	LOS	LOS	LOS	=0%	nil	traction	fixed^t^
8	2816	2756	LOS	3149	LOS	=100%	U(BL)	trauma	fixed^t^
9	1532	3750	LOS	3818	LOS	=100%	L	trauma	fixed^t^
10	729	1395	LOS	1289	LOS	=100%	M	trauma	fixed^t^
11	1748	1784	1998	LOS	LOS	=100%	M	trauma	fixed^t^
12	2154	3823	LOS	3750	LOS	=100%	U(BL)	thermal	fixed^t^
13	1421	1600	LOS	1744	LOS	=100%	M	thermal	fixed^p^
14	2860	3637	LOS	3171	LOS	=100%	U(BL)	thermal	fixed^p^

EMG, electromyography; RLN, recurrent laryngeal nerve; LOS, complete loss of signal; VC, vocal cord; U(BL), upper portion of the exposed RLN (at the region of Berry’s ligament); M, middle portion of the exposed RLN; L, lower portion of the exposed RLN; fixed^ t^, temporary palsy; fixed^ p^, permanent palsy.

Among 105 nerves with a detectable injury site, the weak or disrupted point of nerve conduction was localized at the region of Berry’s ligament (within the upper portion of the exposed RLN) in 83 (79%) nerves (77 traction injuries, 3 dissecting traumas, and 3 thermal injuries), on the middle portion of the exposed RLN in 9 (9%) nerves (8 dissecting traumas and 1 thermal injury), and on the lower portion of the exposed RLN in 13 (12%) nerves (12 dissecting traumas and 1 thermal injury).

## Discussion

When applying IONM in thyroid surgery, the measurement of EMG amplitude from RLN stimulation may correlate with the number of motor units that contribute to polarization and reflect the neurophysiologic function of the nerve (21, 22). Theoretically, unchanged EMG amplitude after RLN dissection indicates normal nerve and VC functions postoperatively. Conversely, decreased EMG amplitude after nerve dissection indicates a nerve function deficit or a decreased number of motor units participating in polarization. Complete LOS after RLN dissection may indicate complete loss of nerve conduction and postoperative VC palsy. However, the correlation between the residual ratio of EMG amplitude after partial RLN injury and the sufficient muscle strength required for normal VC mobility is still unknown.

The intraoperative 4-step procedure (V_1_-R_1_-R_2_-V_2_) has been accepted as a standard IONM procedure by many studies (5, 6, 14–17). It was useful to evaluate nerve function by comparing the EMG signals between pre- and post-dissection of RLN. Unchanged or increased post-dissection EMG amplitudes (comparing R_2_ and V_2_ signals with R_1_ and V_1_ signals, respectively) indicated functional integrity of the RLN. Conversely, decreased R_2_ and V_2_ signals indicated RLN injury and the risk of postoperative VC palsy. However, when using an EMG endotracheal tube for signal recording, unstable EMG amplitudes often occur intraoperatively due to the change in contact quality between the EMG tube and vocal cords. Poor contact quality caused by EMG tube displacement during the surgical maneuver on the trachea will lead to a significant decrease in EMG amplitudes or false LOS. Therefore, a decrease or loss of the post-dissection EMG amplitude would not necessarily be a nerve injury. Genther et al. reported that the risk of immediate postoperative VC palsy is approximately 72% when post-dissection EMG amplitudes are less than 200 µV (23). Pavier et al. reported that the risk of VC palsy is approximately 50% when post-dissection EMG amplitudes were under the value of 280 µV and suggested a staged thyroidectomy (24). The data of these 2 studies showed that the false-positive rate ranged from 28% to 50% and indicated that many patients might receive unnecessary second operations. Calò et al. reported that staged thyroidectomy was performed in 37 patients due to LOS, but 8 (22%) patients had normal VC function after their first operations (25). Melin et al. reported that the second operation was performed in 18 patients with LOS, but 8 (44%) patients were confirmed to have primarily intact vocal cord function (26).

In this study, we used the trans-thyroid cartilage EMG signal recording method (19, 27–29). The EMG amplitude will not be influenced by EMG tube displacement and the elicited signals remain high and stable during the entire course of operation that is important to monitor the actual status of RLN function. Furthermore, we routinely test the proximal and distal ends of the exposed RLN after complete nerve dissection. The procedure helps detect unrecognized partial nerve injury and determine its severity by comparing R_2p_ with R_2d_ signals. This series found that the outcomes of VC function were highly correlated with the severity of nerve injury. In 14 nerves with post-dissection complete LOS, all cases showed fixed VC mobility postoperatively. In 94 nerves with RPDR over 10% (ranging from 13% to 93%), 72 nerves showed symmetric VC mobility where the RPDR was ≤50%. Abnormal VC mobility (weak or fixed VC movement) was only found in the nerves with RPDR >50%. The possibility of postoperative abnormal VC mobility was approximately 14%, 67%, 100%, and 100% among the different RPDR stratifications of 51%-60%, 61%-70%, 71%-80%, and 81-93%, respectively.

A weak or disrupted point of nerve conduction is important neurophysiological evidence of nerve injury. By mapping the exposed RLN, we can localize the injured point and clarify the possible mechanism of nerve injury. In this study, we found that the region of Berry’s ligament is the most common site of nerve injury. Among 105 nerves with an injured point detected, 83 (79%) nerves were injured at this site, including 77 traction injuries, 3 dissecting traumas, and 3 thermal injuries. The injured point was localized at the middle portion of the exposed RLN in 10 (10%) nerves (9 dissecting trauma and 1 thermal injury) and at the lower portion in 12 (11%) nerves (11 dissecting trauma and 1 thermal injury).

**Figure 1:**
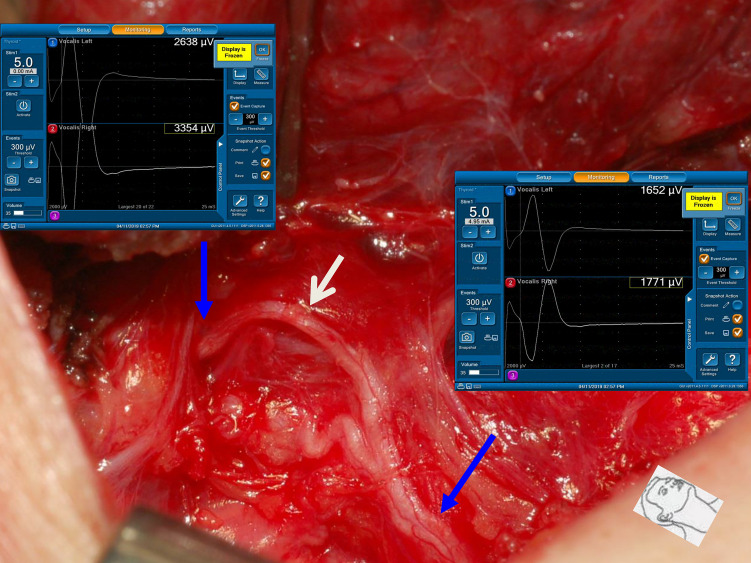
A case of type 1 incomplete LOS. The anterior motor branch of the RLN was stretched upward during medial thyroid traction. After complete RLN dissection and comparing R_2p_ signal (1771 µV) with R_2d_ signal (3354 µV), it showed an amplitude reduction of approximately 47%, and a weak point of nerve conduction was mapped (white arrow).

**Figure 2:**
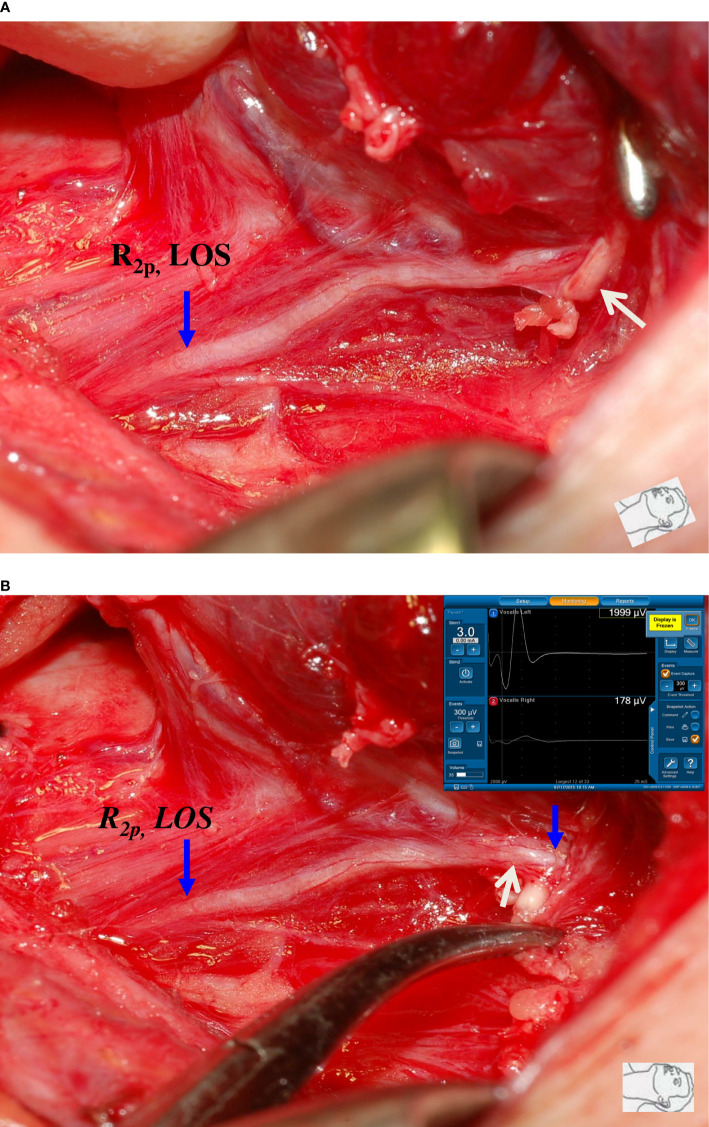
A case of type 1 complete LOS. **(A)** The R_2p_ signal was lost, and a small artery (white arrow) was found to be intertwined with the RLN at the region of Berry’s ligament. **(B)** After dissecting the intertwined vessel from the RLN, R_2d_ signal showed 1999 µV and R_2p_ signal showed complete LOS, a disrupted point of nerve conduction (white arrow) was detected. This was a type 1 LOS and the patient had temporary vocal cord palsy.

**Figure 3:**
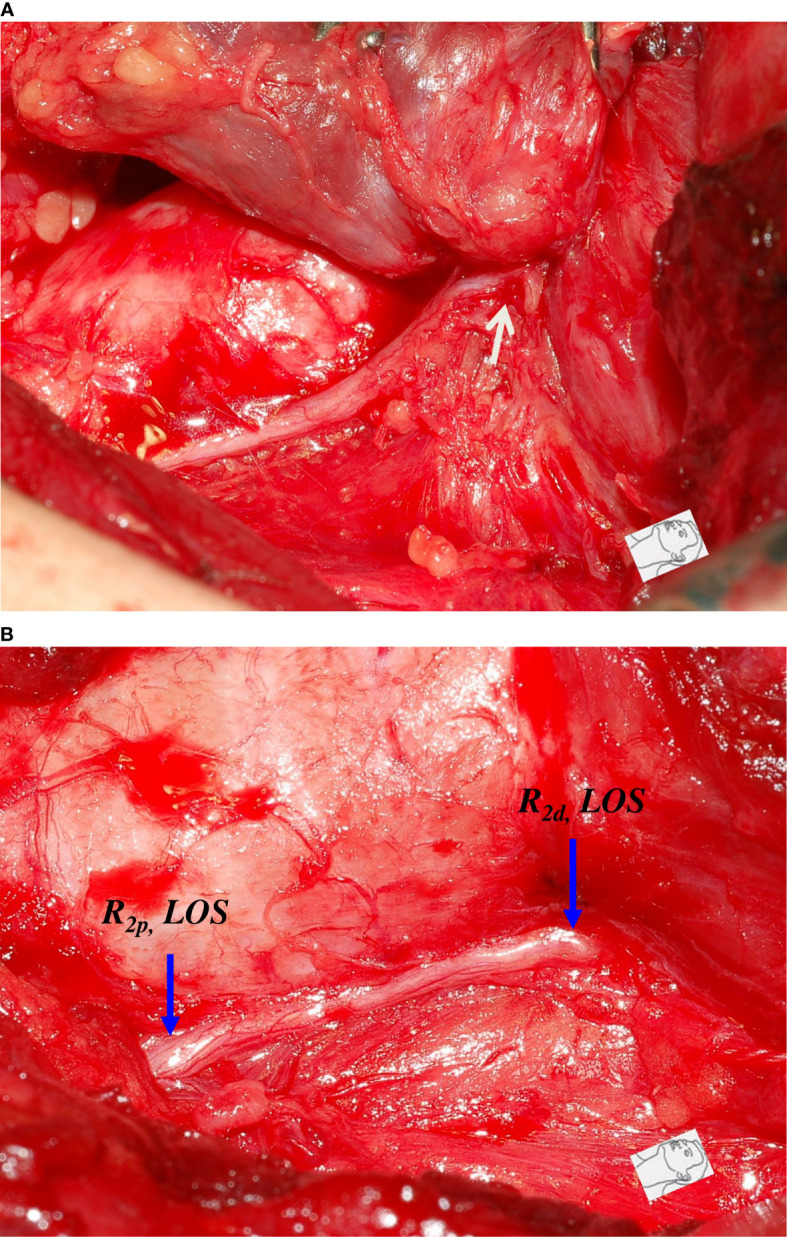
A case of type 2 complete LOS. **(A)** The RLN was stretched upward (white arrow) at the region of Berry’s ligament during medial thyroid traction. **(B)** After complete RLN dissection, both R_2p_ and R_2d_ signals showed complete LOS and no injured point was found on the exposed RLN. This was a type 2 LOS and it developed temporary vocal cord palsy.

**Figure 4:**
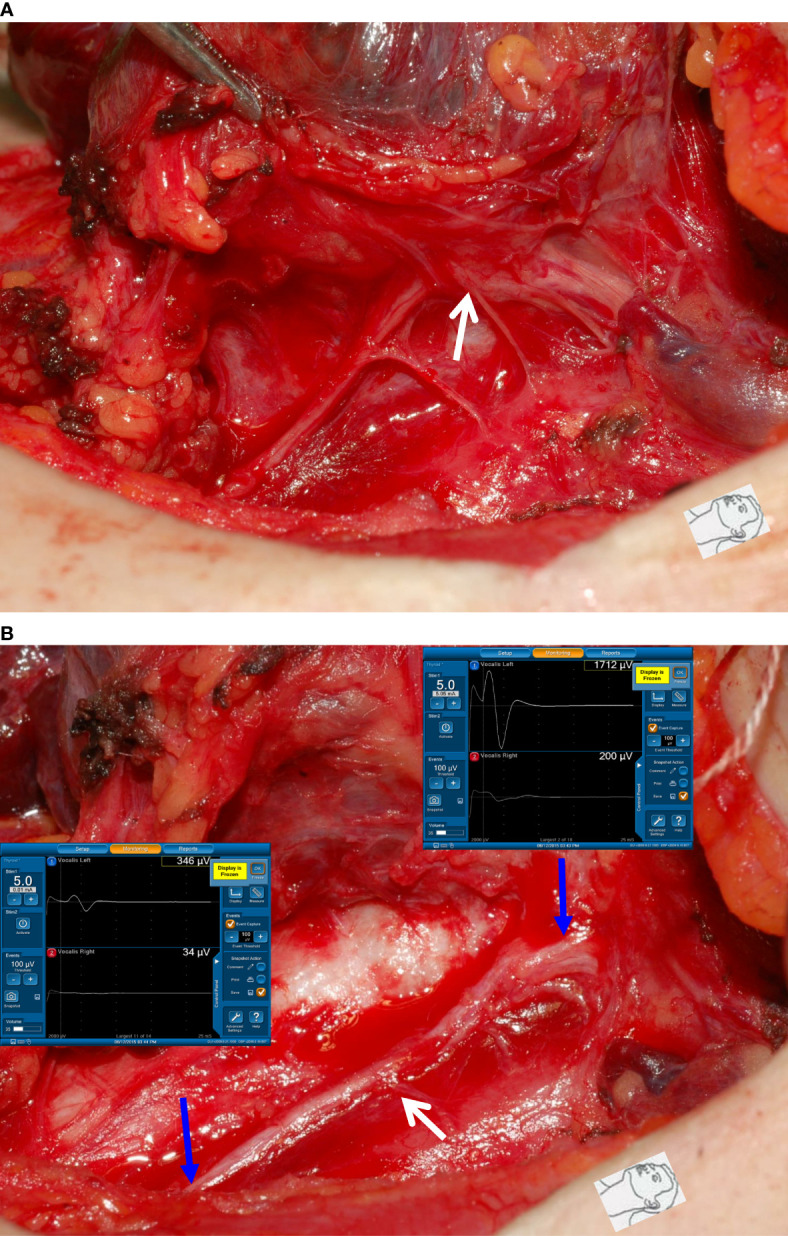
Dissecting trauma of the RLN. **(A)** The RLN was adherent to thyroid cancer. **(B)** After complete RLN dissection and comparing R_2p_ signal (346 µV) with R_2d_ signal (1712 µV), it showed an approximately 80% amplitude reduction and an injured point caused by dissecting trauma was mapped (white arrow). The patient had temporary vocal cord palsy.

**Figure 5:**
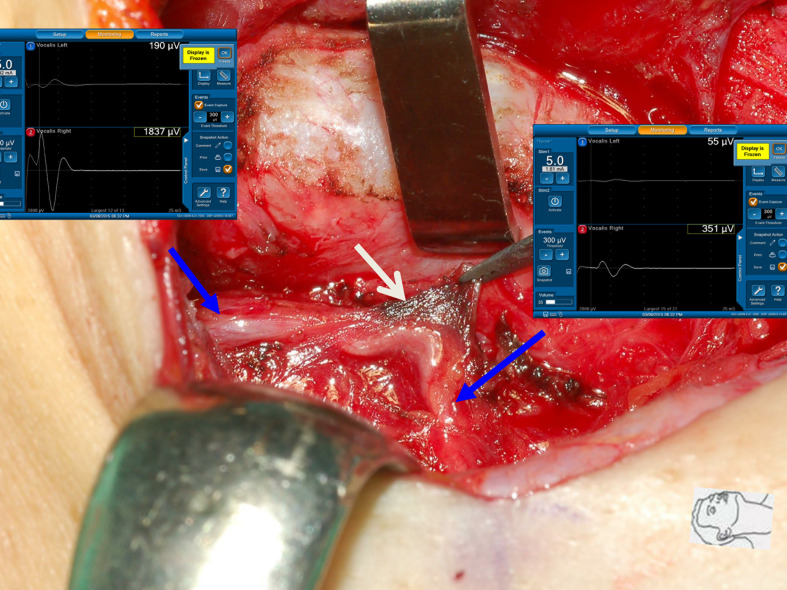
Thermal injury of the RLN with incomplete LOS. The RLN was injured by lateral thermal spread of electrocauterization (white arrow). Comparing R_2p_ signal (351 µV) with R_2d_ signal (1837 µV) showed an approximately 81% amplitude reduction and the development of temporary vocal cord palsy.

**Figure 6:**
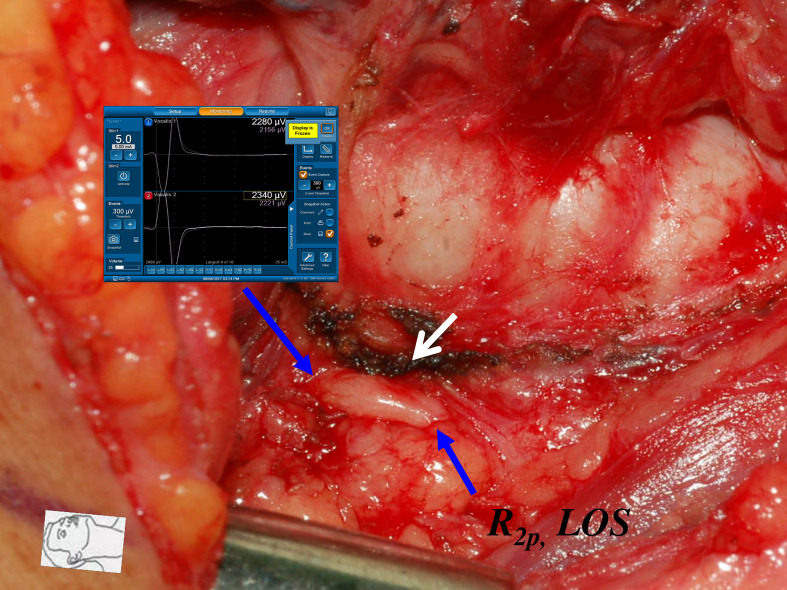
Thermal injury of the RLN with complete LOS. The RLN was injured by lateral thermal spread of energy-based device (white arrow) at the region of Berry’s ligament. Complete LOS occurred in R_2p_ signal, and the R_2d_ signal was 2340 µV. The patient had temporary vocal cord palsy.

This study also found that traction injury was the most common cause of RLN injury, of approximately 74% (80/108). The RLN can be injured at the region of Berry’s ligament by stretching dense fibrous tissue or surrounding blood vessels during medial thyroid retraction. Of the 80 nerves with traction injuries, the injury points were located at Berry’s ligament region in 73 nerves without LOS (**Figure 1**). In the other 7 nerves with LOS, 4 injury points were located at Berry’s ligament region (type 1 LOS) (**Figures 2A, B**), and 3 injury points were not found (type 2 LOS), and the injury point may be located higher above the laryngeal entry point (**Figures 3A, B**). Dissecting trauma was the second most common cause of RLN injury, with a rate of approximately 21% (23/108). Of the 23 nerves injured by dissecting trauma, 3 nerve injuries occurred during dissection of the RLN from Berry’s ligament, 8 nerve injuries occurred during dissection of the RLN from malignant tumors or recurrent benign tumors (**Figures 4A, B**), and 12 nerve injuries occurred during paratracheal node dissection. Thermal injury accounted for approximately 5% (5/108), and all 5 nerve injuries were caused by lateral heat spread of the energy-based device (EBD) or electrocauterization (**Figures 5**, **6**). In 3 nerves with LOS, 2 nerves developed permanent VC palsy, and 1 developed temporary VC palsy. Another nerve with RPDR of 81% developed temporary VC palsy. The other nerve with RPDR of less than 50% showed symmetrical VC mobility after surgery.

From the results of this study, it can be found that permanent VC palsy rarely occurred in nerve injuries caused by traction or dissecting trauma, but there was a high rate of permanent VC palsy (40%, 2/5) in thermal injuries. The voice outcome and VC mobility prognosis are worse in patients with thermal RLN injuries than in those with mechanical RLN injuries (30, 31). Therefore, the use of EBDs or electrocauterization near the RLN should pay special attention to the safety distance. Several studies on animals have shown that activations of various EBDs at 2 mm are safe under electrophysiological evidence (32–34), but translating the data to clinical practice must be very careful. When EBD is activated, tissue contraction can reduce the safety distance and increase the risk of thermal injury. Additionally, hot tissue fluid contact with the RLN may also cause thermal injury (35). Lin et al. confirmed that complete LOS occurred when the RLN was in contact with hot water at 60°C (36). Therefore, we recommend that the RLN should be clearly visualized and that the safety distance from the nerve is best to have 5 mm (37). If the safe distance is less than 5 mm, particularly at the region of Berry’s ligament, a small piece of wet gauze or forceps should be placed between the nerve and the EBD to prevent thermal injury caused by lateral heat spread or by hot tissue fluid. Based on the above, elucidating the mechanism (traction injury, dissecting trauma, and thermal injury) and type (detectable or undetectable injury site) of nerve injury while performing R_2p_ and R_2d_ steps enables thyroid surgeons to continuously improve surgical techniques and reduce surgical complications.

Several limitations should be mentioned in this study. First, this was an observational study without a control group. Second, the number of patients with complete LOS was relatively small; however, the characteristics of severe incomplete LOS and complete LOS can be clearly shown in this study. Last, the functional outcomes and prognosis of type 1 and type 2 LOS warrant further study, and a well-designed study would help to highlight the advantages of R_2p_ and R_2d_ steps.

## Conclusion

Routinely testing the proximal and distal ends of the exposed RLN is a simple and useful procedure to accurately evaluate RLN function after complete dissection. It helps detect unrecognized nerve injury and clarifies where and how the nerve was injured and determines the severity of nerve injury. The provided information will be useful to improve surgeons’ surgical techniques and help make decisions regarding staged thyroidectomy. The additional step of testing the distal end of the exposed RLN should be included in the standard IONM procedure.

## Data Availability Statement

The original contributions presented in the study are included in the article/Supplementary Material. Further inquiries can be directed to the corresponding authors.

## Ethics Statement

The studies involving human participants were reviewed and approved by Institutional Review Board (IRB) of Kaohsiung Medical University Hospital, Taiwan: KMUHIRB-E(II)-20200348. Written informed consent for participation was not required for this study in accordance with the national legislation and the institutional requirements.

## Author Contributions

Supervision – T-ZH, C-WW, GD, and F-YC; Materials – C-FL, T-ZH, Y-CS, T-YH, and F-YC; Data Collection and Processing – H-YH, Chih-CW, Chien-CW, Y-CS; Analysis and Interpretation – H-YH, C-FL, T-YH, and F-YC; Literature Search – H-YH, Chih-CW, Chien-CW, T-YH, and F-YC; Writing Manuscript – All authors. All authors have read and agreed to the published version of the manuscript.

## Funding

This study was supported by grants from Kaohsiung Medical University Hospital, Kaohsiung Medical University (KMUH110-0R51), and Ministry of Science and Technology (MOST 110-2314-B-037-104-MY2, MOST 110-2314-B-037-120), Taiwan.

## Conflict of Interest

The authors declare that the research was conducted in the absence of any commercial or financial relationships that could be construed as a potential conflict of interest.

## Publisher’s Note

All claims expressed in this article are solely those of the authors and do not necessarily represent those of their affiliated organizations, or those of the publisher, the editors and the reviewers. Any product that may be evaluated in this article, or claim that may be made by its manufacturer, is not guaranteed or endorsed by the publisher.
